# Cutaneous Phaeohyphomycosis Caused by *Alternaria alternata* Unresponsive to Itraconazole Treatment

**DOI:** 10.1155/2011/385803

**Published:** 2011-12-18

**Authors:** Joana Gomes, Catarina Vilarinho, Maria da Luz Duarte, Celeste Brito

**Affiliations:** Dermatology and Venereology Department, Hospital de Braga, 4701-965 Braga, Portugal

## Abstract

Cutaneous alternariosis is an opportunistic infection that has been described mainly in patients treated with corticosteroids. We report a case of dermal alternariosis occurring in a woman treated with corticosteroids after a neurosurgery that was unresponsive to itraconazole. Treatment with intravenous voriconazole was initiated, followed by oral protocol, with marked improvement of the lesions.

## 1. Introduction

 Dematiaceous or darkly pigmented fungi are responsible for a wide variety of infectious diseases. They are often found in soil and generally distributed worldwide [1]. *Alternaria* species are the most important causative agents. They are opportunistic fungi that mostly infect immunocompromised patients [2–4]. Most affected individuals are immunodepressed due to Cushing disease, kidney transplants, haematologic malignant diseases, and AIDS [5].

## 2. Case Report

**Figure 1:**
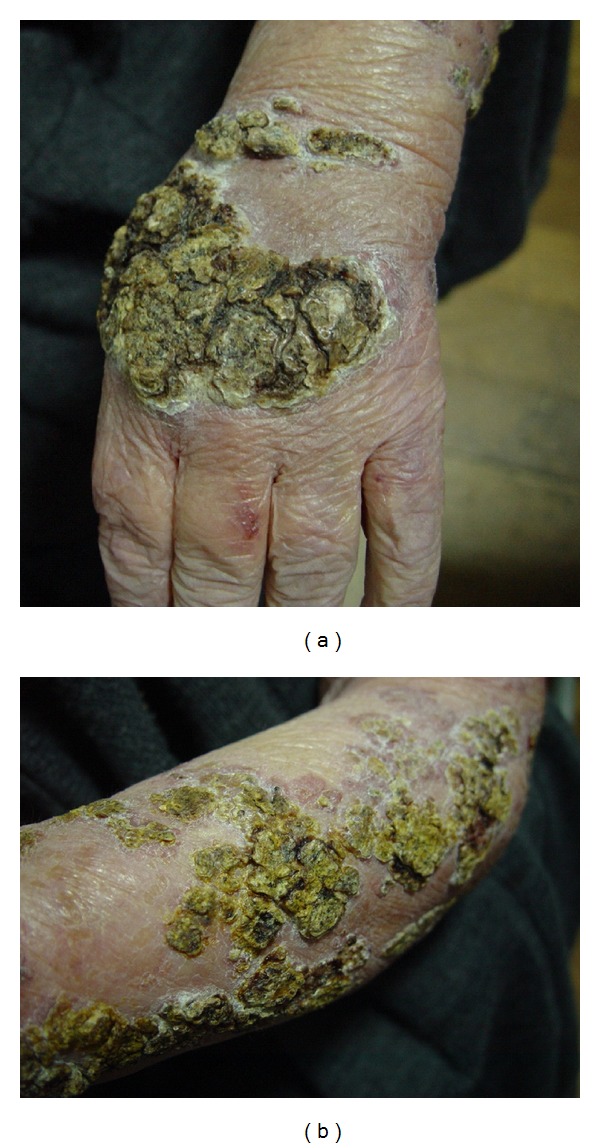
Infiltrating plaques, with a verrucoid appearance, occupying the back of hands (a) and forearms (b).

A 76-year-old woman, resident in a rural area, came to our observation with multiple slowly growing, painless, erythematous infiltrating plaques, with a verrucoid appearance, occupying the back of hands, forearms, and the distal 2/3 of the arms (Figures 1(a) and 1(b)). She had neurosurgery 15 years before and since then she had been treated with 4 mg/day of methylprednisolone orally. The lesions appeared progressively over two years, they were asymptomatic, and there was no history of local trauma.

**Figure 2:**
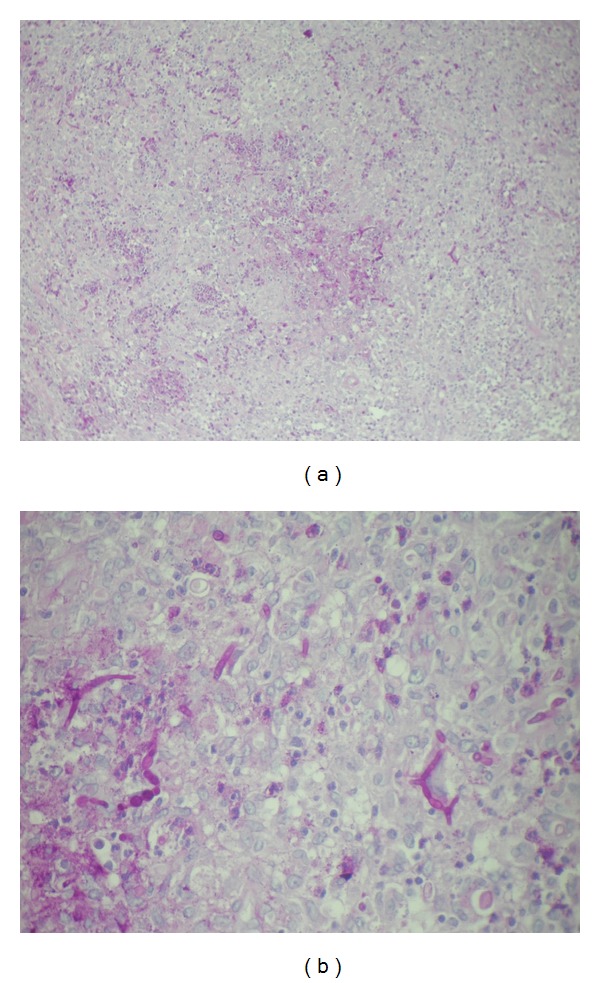
Segmented hyphae and spherical spore-like bodies in the dermis (PAS: (a) 10×; (b) 40×).

**Figure 3:**
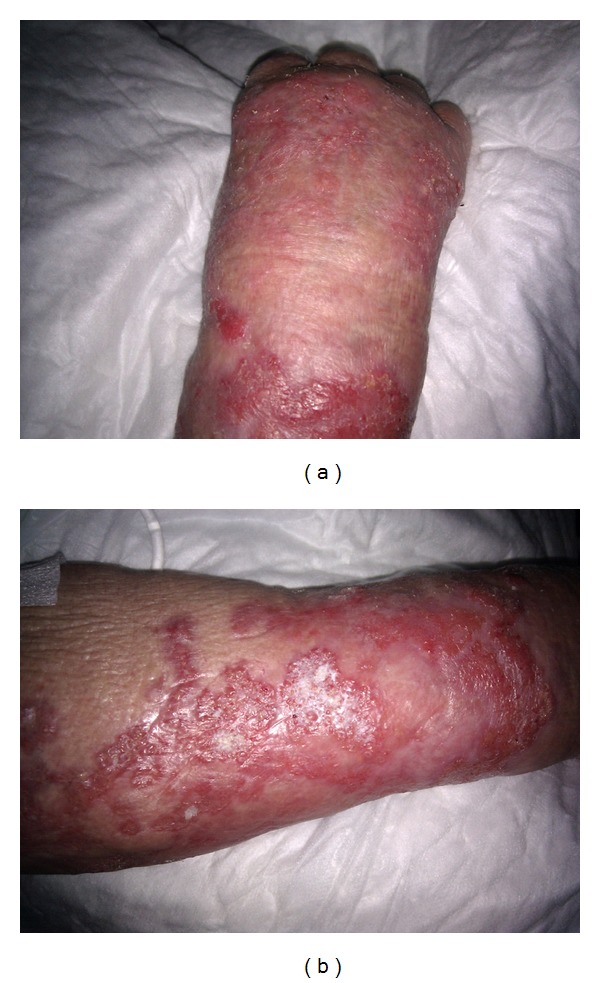
Lesions after 3 weeks of treatment with Voriconazole.

Histopathological examination revealed an intense inflammatory process in the dermis, and several septate hyphae and round or oval spore-like structures that stained positively with periodic acid Schiff stain (Figures 2(a) and 2(b)). Cultures of cutaneous biopsies grew *Alternaria* sp., identified as *Alternaria alternata* by DNA analysis. Due to the extension of the lesions, surgical excision was not feasible. We started itraconazole (100 mg twice daily) and also reduced the corticosteroids, but no improvement was observed after 8 months of treatment. Treatment with intravenous voriconazole at a dose of 4 mg/kg every 12 hours was initiated, followed by 100 mg orally after day 10, with marked improvement of the lesions (Figures 3(a) and 3(b)). However, 4 months after stopping the voriconazole the patient developed congestive heart failure. Her condition deteriorated over the time, and she subsequently died.

## 3. Discussion

Rare molds are increasingly emerging as a cause of deep and invasive fungal infections. Cutaneous alternariosis can no longer be considered rare. Their importance as opportunistic pathogens is increasing, especially in transplant recipients [6]. It has also been described with frequency in patients treated with systemic or local corticosteroids, as in our patient [7–10]. Cutaneous fragility induced by hypercorticism could be an important cofactor permitting direct inoculation from the environment [7, 11]. Our patient lived in a rural area, which could have favored inoculation through a minor trauma.

Dematiaceous fungi may have unique pathogenic mechanisms owing to the presence of melanin in their cell walls. It is thought to be a virulence factor by conferring a protective advantage, scavenging free radicals and hypochlorite that are produced by phagocytic cells and normally kill most organisms [1, 10].

Clinically, most presentations are localized skin infections typically occurring on exposed areas of the body, especially the arms and legs, resulting from traumatic inoculation [1, 2, 5, 6, 12].

Due to their frequent occurrence as laboratory contaminants, *Alternaria* ssp. must be demonstrated by isolation in culture plus histologic evidence of its presence in tissues [5].

Therapy is not standardized given the lack of comparative clinical trials, because these are rare infections. Many antifungals have been used with variable success. Triazoles such as voriconazole, posaconazole, and itraconazole are the most active antifungal agents available [1]. Itraconazole is the preferred drug of choice, because of the lower toxicity and easier administration, and because there is greater clinical experience [12], curiously, in our case itraconazole was ineffective. Surgical excision may be effective, but in our case was not feasible due to extension of the lesions. Voriconazole proved an effective alternative, resulting in disappearance of the lesions. The reason for failure of itraconazole treatment in our patient is unclear. Subtherapeutic tissue concentration at the site of infection is possible. Given the lack of comparative clinical data, decisions over which azole to use will be largely empiric. Voriconazole and posaconazole are generally better tolerated and have broader spectrum of activity.
